# Replication data collection highlights value in diversity of replication attempts

**DOI:** 10.1038/sdata.2017.28

**Published:** 2017-03-14

**Authors:** K. Andrew DeSoto, Martin Schweinsberg

**Affiliations:** 1 Association for Psychological Science, 1800 Massachusetts Ave NW, Ste 402, Washington, District Of Columbia 20036-1218, USA; 2 Department of Organizational Behavior, ESMT Berlin, Schlossplatz 1, Berlin 10178, Germany

**Keywords:** Psychology, Research data

## Abstract

Researchers agree that replicability and reproducibility are key aspects of science. A collection of Data Descriptors published in *Scientific Data* presents data obtained in the process of attempting to replicate previously published research. These new replication data describe published and unpublished projects. The different papers in this collection highlight the many ways that scientific replications can be conducted, and they reveal the benefits and challenges of crucial replication research. The organizers of this collection encourage scientists to reuse the data contained in the collection for their own work, and also believe that these replication examples can serve as educational resources for students, early-career researchers, and experienced scientists alike who are interested in learning more about the process of replication.

## Comment

A fundamental characteristic of science is that scientific claims can be tested. One process through which scientists evaluate the truth of a scientific claim is through the process of *replication*, sometimes called *results reproduction*^1^ (see ref. 2 for a historical introduction). In replication attempts, scientists attempt to repeat a series of experimental methods and ascertain whether the results produced agree or disagree with results of previous research. Scientists, the public, and even policymakers believe that ideally, scientific findings ought to be replicable; in 2015, for instance, the United States House of Representatives noted, ‘The gold standard of good science is the ability of a researcher or research lab to reproduce a published method and finding’^3^.

Concerns have increased, however, that many scientific studies cannot be reproduced or replicated, and that the findings described in some published articles are false (see ref. 4 for a recent estimate of reproducibility in one field). Scientists and philosophers have identified that certain research practices, incentives, and infrastructure challenges may lead to the generation of scientific findings that are not reproducible^5^. These researchers have argued that threats to reproducible science can emerge at all steps of the scientific process, ranging from hypothesis generation to the interpretation of results.

Given that the accuracy of scientific claims made may vary, scientists’ efforts to replicate previous findings become an essential part of science’s self-correcting process. Replication of previous research is thus a cornerstone of the scientific process, in the same way that the generation of new findings and hypotheses helps establish new knowledge. Today, *Scientific Data* launches a collection of papers describing datasets that were generated to help attain that scientific ‘gold standard’ of science—the replication of previous experimental studies (http://www.nature.com/sdata/collections/replicationdata). And as these papers show, despite the wide scientific consensus that indeed replication and reproducibility are key aspects of science, approaches to replication vary.

The call for papers for this special collection invited researchers to submit datasets they had generated in the process of attempting to replicate findings in published papers. Importantly, we, the organizers of this special collection, invited authors to submit datasets belonging to published but also unpublished replication attempts. Publishing datasets belonging to unsuccessful replications is a critical aspect of avoiding the ‘file drawer problem’ in science (e.g., refs 6–8 for recent editorials on this topic). Our hope is that the datasets presented in this collection will assist researchers in developing future research questions and conducting more accurate meta-analyses, and that they will provide proof-of-concept examples that can guide students and researchers in making their work more reproducible.

The replications presented here highlight the diversity of approaches available to scientific investigators when conducting replication science. They also show that many paths lead to the same important end of strengthening replicability. An article by Tierney *et al.*^9^ describes data from a ‘pre-publication independent replication initiative’ (see also ref. 10). Tierney and colleagues describe an attempt to replicate ten lines of research on moral judgment effects that one researcher had in the ‘pipeline’—that is, research that the lab had generated prior to submission to an academic journal. As the authors note, ‘results revealed a mix of reliable, unreliable, and culturally moderated findings’ (p. 1), thus allowing the author to pursue further research on the most robust, reliable effects (while making all results, including the unreliable ones, public). This novel pre-publication replication approach is a way to ensure that one does not accidentally publish a false claim in print or online, when such an error could have been avoided. This format is similar to Registered Replication Reports, in which multiple labs attempt to reproduce a result after publication (rather than before publication) using the same experimental protocol (see ref. 11 for a recent example).

On the other hand, researchers can attempt to replicate research once it has already made it to print—indeed, this is the most common kind of replication: a post-publication, rather than a pre-publication, replication attempt. One example of a post-publication attempt published in this collection is by DuPre and Spreng^12^, which depicts an author’s attempt to replicate his own prior study^13^. This replication, which confirmed the earlier results, also provides a model for how replication research can be conducted.

Another duo of studies, conducted by different groups of researchers under the direction of a principal investigator at the University of Western Ontario, provides another model of replication. Balakrishnan, Palma, Patenaude, and Campbell^14^, and Babcock, Li, Sinclair, Thomson, and Campbell^15^ attempted to replicate several studies examining the relationship between socioeconomic status and decision-making^16,17^. The success of these replication attempts varied, ranging from not confirming the original findings (‘meta-analysis … found no moderating effect,’ ref. 14, abstract) to supporting the original findings (we ‘found a pattern consistent with the original study,’ ref. 15, p. 2). These results and data highlight the challenges inherent to conducting replication work and the difficulties in interpreting the results of even highly-powered replications.

And last, we present replication data from Christmann and Göhring^18^, who successfully replicated results of a 2011 paper on the ways in which metaphors can frame reasoning^19^. This data paper stands out because it describes replication data collected in a different language (German) from the one in which the original study was conducted (English). Christmann and Göhring’s replication data provide evidence that these metaphor effects may extend across culture and language. The new replication attempt also suggests that these metaphor effects generalize to new stimulus sets (e.g., see ref. 20, which showed that certain word length effects do not generalize to other materials).

The papers in this special collection also demonstrate that many online tools, services, and repositories can support scientists in conducting replications. Many of the researchers publishing in this collection used the Open Science Framework (OSF) to manage their workflow. DuPre, Luh and Spreng used Dryad Digital Repository (http://datadryad.org/) and OpenfMRI (https://openfmri.org/; ref. 21) to host their files. Any interested researchers, or members of the public, can access these data files for free. Of course, the papers describing these datasets along with their Data Descriptors published in this special issue are also open and freely accessible. These Data Descriptors are detailed descriptions of the research datasets in question, which include methods used to collect the data and analyses that support the quality of the measurements, aiming to help others reuse data.

Additionally, although we are behavioral scientists, and the work represented here comes from psychology and neuroscience, we believe that the diversity of approaches presented here is of relevance science-wide. These datasets contain important lessons: the importance of replicating one’s own work, both prior to or after publication or both; the value of having other laboratories investigate important original findings; and the question of whether findings will generalize to new stimulus sets and extend to different languages and cultures.

Last, this special collection’s aim of assembling replication studies underlies the important mission of *Scientific Data* as a journal. As the journal’s introductory editorial says, ‘The question is no longer whether research data should be shared, but how to make effective data sharing a common and well-rewarded part of research culture’^22^. Providing an outlet for publication of replication data incentivizes researchers to engage in the critical process of replication. More generally, an outlet for publication of data is likely to increase the proportion of researchers who share their data. This offering supplements other techniques currently used to increase rates of data sharing, such as the Open Data and Open Materials badges awarded by the journals *Psychological Science*, *American Journal of Political Science*, and others, which have been shown to increase rates of data sharing^23^. The more encouragement authors have to share their data, the better, especially when it comes to scientific replications, which have traditionally often gone unrewarded. We continue to encourage authors to submit their replication data to *Scientific Data*.

## Additional Information

**How to cite this article:** DeSoto, K. A. & Schweinsberg, M. Replication data collection highlights value in diversity of replication attempts. *Sci. Data* 4:170028 doi: 10.1038/sdata.2017.28 (2017).

**Publisher’s note:** Springer Nature remains neutral with regard to jurisdictional claims in published maps and institutional affiliations.
